# Perinuclear Lamin A and Nucleoplasmic Lamin B2 Characterize Two Types of Hippocampal Neurons through Alzheimer’s Disease Progression

**DOI:** 10.3390/ijms21051841

**Published:** 2020-03-07

**Authors:** Laura Gil, Sandra A. Niño, Erika Chi-Ahumada, Ildelfonso Rodríguez-Leyva, Carmen Guerrero, Ana Belén Rebolledo, José A. Arias, María E. Jiménez-Capdeville

**Affiliations:** 1 Departamento de Genética, Escuela de Medicina, Universidad “Alfonso X el Sabio”, 28691 Madrid, Spain; lgilalb@uax.es (L.G.);; 2 Departamento de Bioquímica, Facultad de Medicina, Universidad Autónoma de San Luis Potosí, San Luis Potosí 78210, Mexico; 3 Servicio de Neurología, Hospital Central “Ignacio Morones Prieto”, San Luis Potosí 78290, Mexico; 4 Banco de cerebros (Biobanco), Hospital Universitario Fundación Alcorcón, Alcorcón, 28922 Madrid, Spain

**Keywords:** Alzheimer’s disease, Tau protein, Lamin A, Lamin B2, heterochromatin, cell-cycle, hippocampus, neurofibrillary tangles

## Abstract

Background. Recent reports point to a nuclear origin of Alzheimer’s disease (AD). Aged postmitotic neurons try to repair their damaged DNA by entering the cell cycle. This aberrant cell cycle re-entry involves chromatin modifications where nuclear Tau and the nuclear lamin are involved. The purpose of this work was to elucidate their participation in the nuclear pathological transformation of neurons at early AD. Methodology. The study was performed in hippocampal paraffin embedded sections of adult, senile, and AD brains at I-VI Braak stages. We analyzed phospho-Tau, lamins A, B1, B2, and C, nucleophosmin (B23) and the epigenetic marker H4K20me3 by immunohistochemistry. Results. Two neuronal populations were found across AD stages, one is characterized by a significant increase of Lamin A expression, reinforced perinuclear Lamin B2, elevated expression of H4K20me3 and nuclear Tau loss, while neurons with nucleoplasmic Lamin B2 constitute a second population. Conclusions. The abnormal cell cycle reentry in early AD implies a fundamental neuronal transformation. This implies the reorganization of the nucleo-cytoskeleton through the expression of the highly regulated Lamin A, heterochromatin repression and building of toxic neuronal tangles. This work demonstrates that nuclear Tau and lamin modifications in hippocampal neurons are crucial events in age-related neurodegeneration.

## 1. Introduction

The nuclear lamin (NL) is a structural framework of four intermediate filaments type V composed by a peripheral nuclear lamina and a diffuse internal network [1] anchored to the nucleoskeleton [2]. Two lamin subtypes, A and B, each of them with different physicochemical properties and cell type-specific expression levels, are their main components [3]. The human gene that codifies the Lamin A subtype, *LMNA*, displays alternative splicing giving rise to the isoforms Lamin A and Lamin C, while two different genes *LMNB1* and *LMNB2* codify for Lamin B1 and B2, respectively [4]. From its location adjacent to the inner nuclear membrane, NL extends through the nucleoplasm regulating chromatin dynamics and providing nuclear structural scaffolding [5,6]. The complex relationship between NL and chromatin specializes in the modulation and maintenance of euchromatin and heterochromatin domains or chromosomic territories. It regulates DNA and RNA synthesis by building a replication and transcription scaffold, and it orchestrates the DNA damage response, safeguarding genome stability [7,8,9,10]. More than 40% heterochromatin is associated with the nuclear periphery, forming the lamin-associated chromatin domains (LADs) and to the nucleolar periphery, defined as the nucleolus associated domains (NADs) [11,12]. The LADs and NADs contain most of the heterochromatin from centromeric and pericentromeric chromosomal regions and they are enriched in repressive chromatin marks such as H3K9me2/3 and H4K20me3 [13,14]. Therefore, they can exchange their positions according to Lamin A, B1, and B2 levels [15] determining the nuclear configuration under diverse environmental conditions [11]. 

Alzheimer’s disease (AD) is the most prevalent neurodegenerative disorder associated with aging. Although Tau protein hyperphosphorylation and aggregation are hallmarks associated with the progress of the disease [16], recent studies point to a nuclear origin of AD, whereby nuclear Tau [17,18], and NL proteins [19,20] would play a decisive role. Tau interacts with DNA [21,22,23], including heterochromatin contacts [24,25], genome protection [26,27,28,29] by modulating euchromatin gene expression [30,31] and it accumulates in cell nuclei throughout neuronal aging [32]. AD onset and progression takes place in parallel with the gradual disappearance of Tau from neuronal nuclei and its accumulation in the cytoplasm [17]. In *Drosophila melanogaster* these changes are coupled to NL dysfunction [33] and epigenetic modifications of global chromatin such as variations of H3K9me2/3, HP1alpha, H3K9ac, and H3K12ac [1,19,34]. Nucleolar chromatin displays hypermethylation of rDNA genes [35]. All these phenomena are linked to the aberrant cell cycle re-entry that aged neurons initiate [36]. Heterochromatin modifications, in turn, result in decreased rRNA synthesis and induce nucleolar stress, affecting ribosome number, protein transduction, and gene expression [37,38,39]. In the nervous system, Lamins A/C, B1, and B2 present different expression levels and are instrumental for embryonary development and postnatal survival [40]. While B1 and B2 alterations are involved in various neuropathies, both in humans and mice [41,42], the absence of Lamin A has not been related to abnormalities [43], given that its expression level in the adult brain is scarce. It is absent in neurons from the mouse cortex, for instance, and Lamin C is expressed in all neuronal types. In this respect, recent research performed in a transgenic *Drosophila* model of tauopathy has demonstrated that Lamin B is implicated in AD onset [33] and suggests that AD could be considered an acquired laminopathy associated to aging [20]. Accordingly, significant alterations of lamin gene expression have been recently reported, which mainly affect the *LMNA* gene and during late AD stages [44]. This work searched for possible alterations of different lamin subtypes in CA1 and CA3 hippocampal regions in autopsied human tissue obtained along I-VI Braak stages. Given the critical role of nuclear Tau in pericentromeric heterochromatin repair [25], we analyzed lamin and heterochromatin changes through the specific histone marker H4K20me3 in the human hippocampus. We hypothesized that the abnormal cell cycle reentry associated with early AD stages implies a dramatic transformation of the quiescent nucleoskeleton (G0) of postmitotic neurons. This transformation is implemented in order to acquire a configuration of maximal transcriptional activity, especially nucleolar (G1) and replicative (S) activities. The creation of this aberrant cycling structure leading to the pathological AD neuron is enabled by fundamental nuclear proteins such as nuclear Tau and Lamins. 

## 2. Results

### 2.1. Phosphorylated Tau Shifts from Nucleus to Cytosol through AD Progression

**Figure 1:**
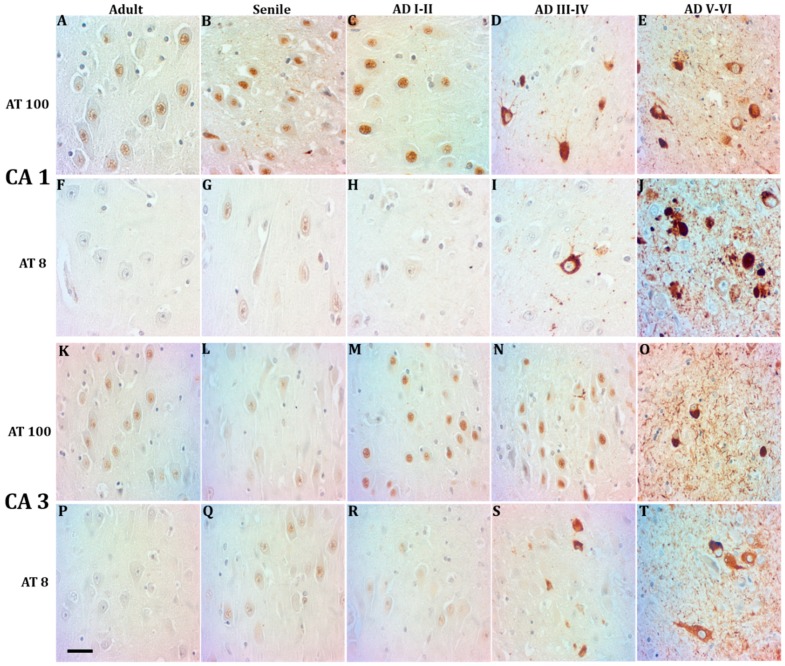
Neurons containing phosphorylated Tau in the CA1 and CA3 hippocampal regions in mature, elderly, and AD I-VI stages of Braak. Immunopositive AT100 nuclei in mature and senile neurons (**A**,**B**,**K**,**L**). AD I-II nuclei present both intense and fading immunostaining (**C**,**M**), while in middle AD stages it shows cytoplasmic and only some slightly positive nuclei (**D**,**N**). Exclusive localization in the cytoplasm in late AD (**E**,**O**). AT8 is present in nuclei of senile and early AD (**G**,**H**,**Q**,**R**) but not in mature stages (**F**,**P**). Tangles and immunopositive neuropile in the middle and late AD stages (**I**,**J**,**S**,**T**). scale bar10 µm.

Two Tau forms were assayed in CA1 and CA3 hippocampal neurons along AD stages and compared with tissue from healthy controls, AT100 and AT8. We confirm here the previously reported fact that AT100 is found in the nuclei of adult individuals (Figure 1A,K) and its expression level increases in CA1 from aging healthy adults (Figure 1B), while at AD I-II stages a mixture of intense and weak nuclear AT100 expression is noticed (Figure 1C,M). Through AD stages III-VI, AT100 immunostaining was gradually vanishing from nuclei, to reach a total cytoplasmic and extracellular localization at AD V-VI stages (Figure 1D,E,N,O). Nuclear AT8 expression, by contrast, was only present in elderly adults (Figure 1G,Q) and scarcely at early AD stages (Figure 1H,R). From AD stages III-VI, AT8 immunopositivity was observed extranuclear in the form of tangles and progressing towards the intensely positive neuropile, extracellular plaques, and ghosts that characterize AD late stages (Figure 1I,J,S,T).

**Figure 2:**
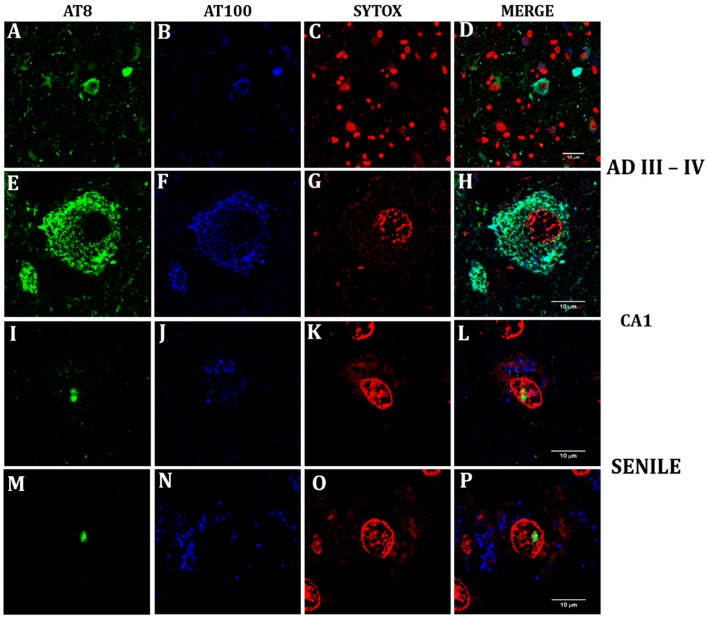
Confocal analysis of nuclear and cytoplasmic phosphorylated Tau in senile CA1 hippocampal neurons and at AD III-IV Braak stages**.** Colocalization of two phosphorylation sites (S212-T214) and (S202-T205) was tested with AT100 and a rabbit monoclonal antibody (AH36), which recognizes the same site as AT8 antibody. The term AT8 was kept in order to follow in line with the previous figure. View of CA1 region at AD III-IV showing the characteristic tauopathy through AT100 (blue), AT8 (green) and nuclei stained in red with SYTOX (**A**–**D**). Detail of a neurofibrillary tangle with cytoplasmic AT100 (**E**) and AT8 (**F**). Nuclear presence of the site recognized by AT8 (**I**,**M**), and of AT100 site (J) in a neuron from senile hippocampus. AT100 was also identified in the cytoplasm of senile neurons (**N**,**P**). Senile CA1 neuronal nuclei can simultaneously express AT8 and AT100 (**L**), or nuclear AT8 and cytoplasmic AT100 (**P**).

Figure 2 shows the confocal immunofluorescence imaging of AT100 (blue) and AT8 (green). The classical view of tangles, neuropile, and extracellular cumuli marked with the AT8 and AT100 antibodies is shown in a section through CA1 from a hippocampus at AD stages III-IV (Figure 2A–D). A closer look at the neurofibrillar tangle displays the simultaneous presence of both phosphorylated Tau forms (Figure 2E,F) and their absence in the neuronal nucleus (Figure 2G,H). By contrast, their localization is nuclear in senile neurons from a healthy hippocampus (Figure 2I,J) in contact with nuclear material (Figure 2K), while some neurons show only AT8 intranuclear (Figure 2M) and cytoplasmic AT100 (Figure 2N,P). 

### 2.2. Hippocampal Neurons Express Lamin A from Early to Late AD Stages

**Figure 3:**
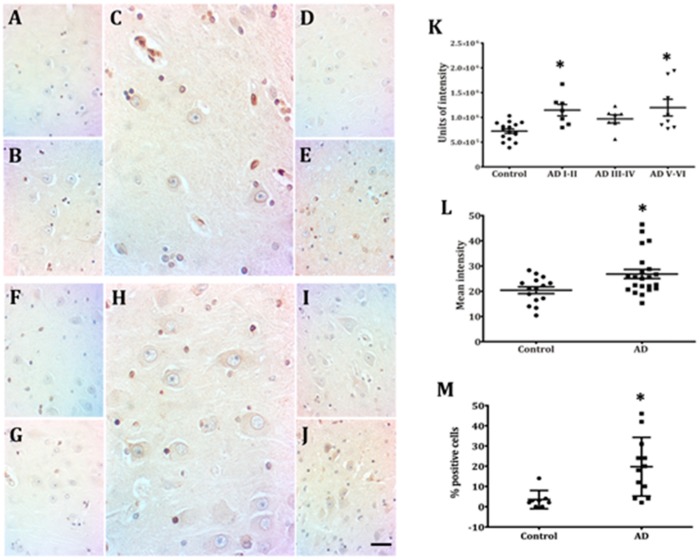
Presence of Lamin A immunopositive pyramidal neurons in CA1 and CA3. Neurons from adult (**A**,**F**) and elderly (**B**,**G**) subjects lack immunopositivity to Lamin A. Increased membrane expression of Lamin A characterizes early AD stages (**C**,**H**) and the presence of immunopositivity continues at late AD stages (**D**,**E**,**I**,**J**), 40X microphotographs, scale bar—10 µm. Quantification of immunopositivity. Significant increases of Lamin A in total intensity (**K**), percentage of positive cells (**L**), and mean intensity (**M**). Each point represents the image analysis of 50 cells per subject in CA1 and CA3 regions. Graphs express mean ± SD, * *p* < 0.05. See the text for further details of image analysis and statistics.

Although Lamin A expression was noticed in other cell types along all analyzed conditions, hippocampal neurons from healthy individuals lacked Lamin A immunopositivity CA (Figure 3A,B,F,G). AD onset was accompanied by a significant increase of neurons expressing Lamin A (Figure 3C,H). The intensity of immunolabeling per cell (Figure 3K), the percentage of positive cells (Figure 3L), and the mean intensity of immunostaining (Figure 3M) increased significantly from early AD stages in both CA1 and CA3 regions. This increased membrane expression remained higher than in healthy controls at all AD stages (Figure 3K). As no difference between CA1 and CA3 was noticed through this technique (Figure 3D,E,I,J), quantification data from both regions were pooled. Contrary to the notorious appearance of Lamin A at early AD stages, no sign of the presence of Lamin C was detected in human pyramidal neurons in any of the analyzed conditions (Supplementary Figure S1). 

### 2.3. Lamin B2 Redistributes in the Nucleoplasm along AD Stages

**Figure 4:**
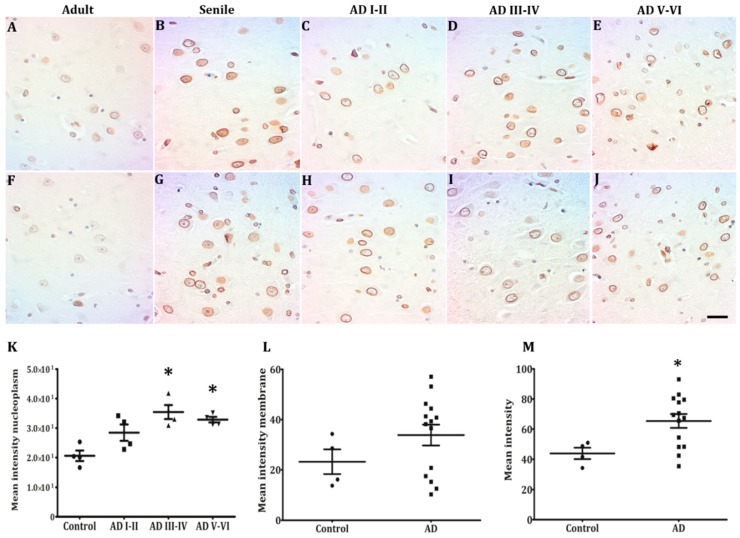
Lamin B2 in pyramidal hippocampal neurons. Lamin B2 immunopositivity in adults is mostly perinuclear (**A**,**F**) and starts appearing in the nucleoplasm in senile subjects (**B**,**G**). Pyramidal neurons in both CA1 and CA3 present either intense perinuclear immunopositivity or homogeneous staining over the nucleoplasm (**C**–**E**,**H**–**J**), 40× microphotographs, scale bar—10 µm. Quantification of immunopositivity. Lamin B2 immunostaining in nucleoplasm increased significantly in the middle and late AD stages (**K**,**M**). The intensity of the membranes clearly reveals two populations of neurons (H, high and low intensity), and Lamin B2 mean intensity increased significantly in AD (**L**). Each point represents the image analysis of 50 cells per subject in CA1 and CA3 regions. Graphs express mean ± SD, * *p* < 0.05. See the text for further details of image analysis and statistics.

**Figure 5:**
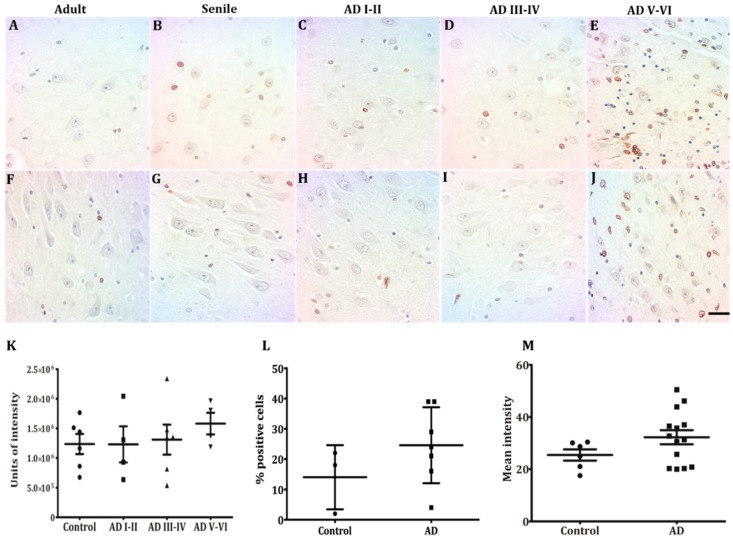
Lamin B1 in pyramidal hippocampal neurons. Low-intensity Lamin B1 immunopositivity is present at all conditions studied (**A**–**J**), with slightly higher levels at AD V-VI (**E**–**J**). Low-intensity Lamin B1 immunopositivity is present at all conditions studied (A–J), with slightly higher levels at AD V-VI, oligodendrocytes and microglia intensely stained (**E**,**J**). Lamin B1 did not present quantitative changes across the different conditions (**K**-**M**). Each point represents the image analysis of 50 cells per subject in CA1 and CA3 regions. Graphs express mean ± SD, * *p* < 0.05. See the text for further details of image analysis and statistics.

The most prominent immunopositivity among hippocampal neurons was Lamin B2 (Figure 4A–J,M). Along with health conditions and AD stages, neurons were highly positive. Both in CA1 and CA3, the highest percentage of immunopositive neurons was found at ADIII-IV stages (Figure 4K). A notorious change observed in Lamin B2 was its distribution in the nucleoplasm. Starting in senile subjects and increasing in early AD stages, Lamin B2 immunopositivity included the whole nucleoplasm of several hippocampal neurons (Figure 4B–D,G–I). The mean intensity in the nucleoplasm almost doubled at intermediate and late AD stages as compared with controls (Figure 4K). The data of mean intensity in the membrane allowed to distinguish two populations (Figure 4L), one with values higher than controls and the other with lower values. In contrast, Lamin B1 immunostaining was always limited to the membrane both in healthy and AD conditions and only presented a slight increase at AD V-VI stages (Figure 5A–J), which did not reach statistical significance (Figure 5K–M).

### 2.4. Nuclear Lamin and Nuclear Tau Changes are associated with Constitutive Heterochromatin Modifications

**Figure 6:**
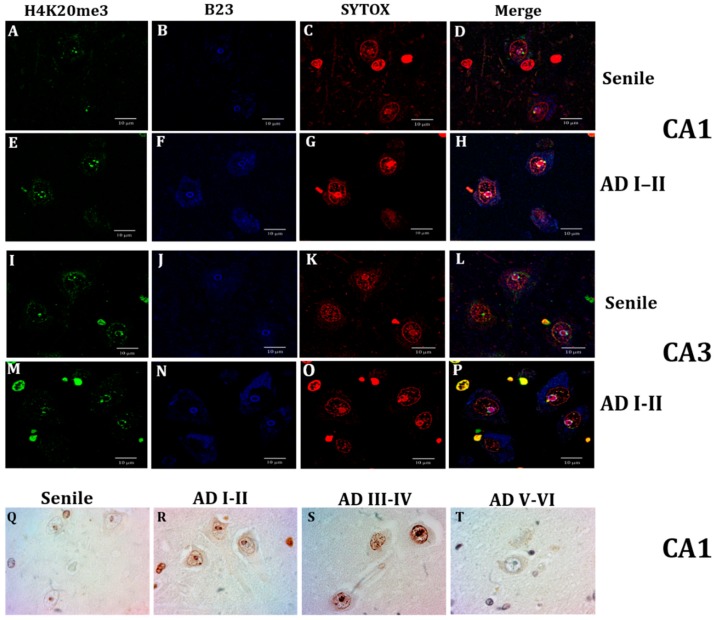
Confocal analysis of H4K20me3 (green), and nucleoli immunofluorescence through nucleophosmin antibody (B23, blue) and nucleic acids through SYTOX (red). Neurons from a senile subject present in CA1 (**A**) and CA3 (**I**) scarce positive green marks, and well-delimited nucleoli (**B**,**J**), and the epigenetic H4K20me3 marks localized in the nucleolar chromatin (**C**,**D**,**K**,**L**). A marked increase of nuclear speckles and spots around the nucleoli and adjacent to the nuclear lamina is observed at AD (I-II) (**E**,**M**) and B23 immunofluorescence is not limited to the nucleoli but also dispersed in the cytoplasm (**F**,**N**,**H**,**P**), and H4K20me3 marks are not only in the nucleolar chromatin (**G**,**O**) but also adjacent to the nuclear lamina (**H**,**P**). H4K20me3 immunostaining. Distribution of H4K20me3 immunopositivity around the nucleolus (NADs) and adjacent to the nuclear lamina (LADs) (**Q**). Intensely marked nuclei at AD I-II stages (**R**) and AD II-IV stages (**S**) and null to slight positivity at late AD stages (**T**,**L**), scale bar—10 µm.

Figure 6 displays the nucleolus marker B23 (nucleophosmin, blue) and its close relationship with the heterochromatin marker H4K20me3 (green) in hippocampal CA1 and CA3 neurons. H4K20me3 showed scarce positivity in senile subjects, which was limited to 1 or 2 spots in the nucleolus periphery and, in some cases, a well-delimited spot in the proximity of the inner nuclear membrane (Figure 6A,D,I,L). Early AD stages were characterized, in contrast, by a remarkable increment of H4K20me3 immunopositivity around the nucleolus, in the nucleoplasm and in spots aligned with the inner nuclear membrane (Figure 6E,H,M,P). To better document this important epigenetic modification along AD stages, Figure 6R,S shows the condition of high immunopositivity that remained along AD I-IV stages and decreased almost to control levels at late AD stages (Figure 6T).

### 2.5. Lamin A Expression in Cells with Perinuclear Lamin B2

**Figure 7:**
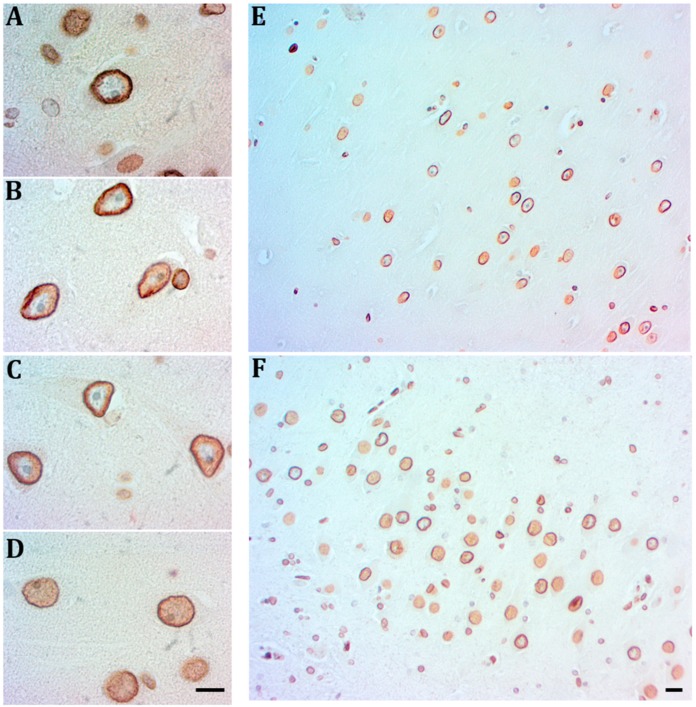
Relationship of Lamin B2 with Lamin A at early AD stages. Lamin B2 immunopositivity (DAB-brown) covers the nucleoplasm of neurons with nucleoli displaced to the periphery or without nucleoli and signs of degeneration (**A**,**D**). A reinforced Lamin B2 associated with Lamin A (red- amino-ethyl-carbazole) characterizes neurons with euchromatic nuclei with prominent nucleoli (**B**,**C**). In the hippocampus at AD I-II stages the two neuron populations coexist in CA1 (**E**) and CA3 (**F**), scale bar—5 µm.

The emergence of B2 positive nucleoplasms signals the senile and early AD stages. At the AD I-II stages, a significant increase of B2 immunopositivity both in the membrane and in the nucleoplasm of numerous pyramidal neurons is associated with nucleoli displaced to the periphery and signs of neuronal attrition (Figure 7A,D). Those cells show either a thin and discontinue Lamin A or no immunopositivity to Lamin A. By contrast, cells expressing a robust Lamin A at the inner side of the nuclear membrane do not show signs of nucleolar displacement and Lamin B2 is limited to the membrane (Figure 7B,C). Both types of cells uniformly occupy CA1 and CA3 regions at early AD stages (Figure 7E,F). 

## 3. Discussion

This work analyzes for the first time the four types of nuclear lamin in postmitotic human hippocampal neurons, its relationship with two forms of phosphorylated nuclear Tau and with constitutive centromeric and pericentromeric heterochromatin. The comparisons were performed in autopsied tissues containing CA1 and CA3 hippocampal regions of healthy controls and AD patients from stage I to VI according to the Braak and Braak classification. 

### 3.1. Neurons Express Lamin A from Early AD Stages on

Our results confirm the absence of Lamin A in postmitotic neurons of the human brain, in agreement with previous reports in mouse brain [45], rat brain [46], and rat retinal neurons [47]. Hippocampal neurons also lack the expression of Lamin C, which is controversial as it has been previously reported in mice and rats [45,46]. These reported findings were confirmed in this study both in *wild type* and triple transgenic AD mice (Supplementary Figure S1). Intriguingly, adult human neurons require only Lamin B1 and B2 to conform to their NL, while low Lamin A expression is individually regulated by miR-9, a brain-specific microRNA [42,45]. In this respect, it has been found that each cellular type assembles varied proportions of lamin subtypes to achieve different levels of mechanic stability according to their specific functions [48,49]. Accordingly, the absence of Lamin A in soft tissues like the brain would favor a flexible nucleus able to interconnect both with the cytoskeleton and with the extracellular matrix [50,51]. 

The most outstanding result of this work is the expression of Lamin A in NL of hippocampal neurons of AD patients. A higher number of immunopositive cells, as well as an increased positive area, where evident at early AD stages. Dynamically modified expression levels of Lamin A have been reported to take place in response to extrinsic mechanisms (stiffness of extracellular matrix and nucleo-cytoskeletal stress) to protect the genome and to regulate the cell-cycle [52,53]. This suggests that the anomalous Lamin A expression at early AD stages could be directly associated with excessive DNA damage and the aberrant cell-cycle reentry of hippocampal post-mitotic neurons [33,44,54,55]. In this context, it could also be speculated that the anomalous Lamin A presence is related to the neuropathology or cognitive deficit [56] as in the rare premature aging disorder of Hutchinson-Gilford progeria syndrome, neurons do not suffer neurodegeneration because they do not express the mutated Lamin A precursor [57].

### 3.2. Lamin B2 in the Nucleoplasm of AD Neurons 

Lamin B1 and B2 are separately assembled, but they interconnect their lattice of fibrils both in the NL and in the nucleoplasm to build a scaffold for heterochromatin [6]. Both molecules are present in human hippocampal neurons as well as in rat cerebral cortex [46] and retinal neurons [47] bearing similar expression levels [44]. A significant reduction of B-type lamin expression and morphologic alterations has been reported in cortical neurons of AD patients as well as in a human Tau transgenic *Drosophila* model [19].

Lamin B1 is fundamental for the assembling of lamins in the NL [6], for the spatial conformation of chromosomic territories, including the nucleolus [58] and the organization of transcription factories in the nucleoskeleton [59,60]. Our results indicate minimal changes in B1 immunopositivity, only a slight increase in AD V-VI stages. By contrast, two remarkable modifications of the expression pattern of Lamin B2 in elderly controls and across all AD stages were observed. While some neurons present a perinuclear reinforced Lamin B2, in other neurons Lamin B2 was distributed in the nucleoplasm (Figure 3C,D,H,I). 

AD brains suffer a significant and selective neuronal loss accompanied by progressive synaptic impairments in the surviving neurons affected by the toxic accumulation of Tau aggregates, especially in the hippocampus [61]. Both events have been related to the exit from the quiescent state of postmitotic neurons and their anomalous cell-cycle reentry in response to DNA damage [55,62]. It is well documented that mitotic cycle proteins are abundant and anomalously expressed in the hippocampus of elderly controls, patients diagnosed with mild cognitive impairment, and AD [63,64,65]. Nevertheless, a significant difference distinguishes the neurons from elderly controls from those of AD patients. Senile neurons are not able to progress further than G1, because they express cyclin D, but not cyclin A [66]. On the other hand, neurons from the mild cognitive deficit, and AD patients do express cyclin B and proliferating cell nuclear antigen, which allows them to replicate their genome. In this way, they became tetraploids and remain stuck in G2-M, unable to undergo mitosis [63,64,67,68]. Relating this concept of “vulnerable neurons” to our results we hypothesize that neurons expressing nucleoplasmic Lamin B2 have entered G1 and are preparing to progress to S phase, based in the chromatin organizing role of Lamin B2 during replication, where it redistributes among the replication factories of the nucleoskeleton [69,70,71,72]. Neurons with reinforced Lamin B2, by contrast, are not able to progress further than G1. It is critical to underline that, although both neuronal types are present in elderly controls and in AD patients, the fact that draws the frontier between aging and neurodegeneration is the appearance of Lamin A in AD neurons stuck in G1 [36,73]. The reinforcing effect of Lamin B2 on the B1 scaffold in early AD neurons modify the NL leading to nucleo-cytoskeleton stress [51], which induces Lamin A expression to protect the genome [52]. Lamin A can also activate DNA repairing programs and regulate the cell cycle [51,53]. A deeper study about the functional implications of perinuclear Lamin A and nucleoplasmic Lamin B2 could be performed using living primary hippocampal neurons cultures where cell cycle can be exogenously triggered [74]. In this work, we confirmed the presence of perinuclear Lamin A and nucleoplasmic Lamin B2 in CA1 neurons of a triple transgenic mouse model (3 × Tg AD model) at the age of 6 months, which corresponds to an early disease stage (Supplementary Figure S2).

### 3.3. Lamin A Associated Epigenetic Changes at Early AD Stages

Replicative aging implies a decrease of the epigenetic marker H3K9me3, which maintains the compacted status of LAD chromatin regions [75]. In postmitotic neurons, however, aging-associated epigenetic regulation is not entirely understood, and this chromatin marker has been found repressing dozens of cell cycle genes [76]. Several reports demonstrate that epigenetic regulation is also driven by changes in the expression levels of lamins [2,77]. In this respect, a decrease of Lamin B and H3K9me3 has been found in the *Drosophila* AD model [33]. Accordingly, our results demonstrate that in addition to Lamin B2 modifications, the anomalous Lamin A expression in pyramidal neurons leads to the up-regulation of H4K20me3, which is also one of the epigenetic markers of HGPS [78]. H4K20me3 is a specific marker of constitutive heterochromatin, whose increased expression is associated with the loss of peripheral or total heterochromatin in HPGS and other laminopathies [79,80,81]. Nevertheless, its markedly increased expression at early and intermediate AD stages leads us to consider the possibility of an essential role in genome stability, to suppress transcription and recombination of repetitive sequences of constitutive heterochromatin induced by nuclear stress [13,80,82,83,84].

### 3.4. Nuclear AT8 in Aging Cycling Neurons

Tau presence in neuronal nuclei has been repeatedly reported over the last three decades [18], but its interaction with genic and intergenic DNA sequences of global chromatin has just recently been elegantly demonstrated [31]. This interaction strongly suggests a role in genome organization, which would define its participation in aging and AD [32,85]. The intrinsically disordered Tau structure undergoes multiple conformational changes upon phosphorylation, which define its function [86]. Among them, AT100 (Tau phosphorylated at Thr212-Ser214 epitope) interacts with global chromatin, progressively increases its nuclear expression with aging, and gradually disappears from the nucleus as AD progresses [17]. This work shows for the first time the nuclear presence of AT8 immunopositivity (Tau phosphorylated at Ser202-Thre205) in aging hippocampal neurons already positive for AT100 (Figure 2). The nuclear coexistence of several Tau phosphorylated forms has also been found in other neurons (unpublished results). AT100 and AT8 are emblematic antibodies employed for the postmortem diagnosis of AD because they are markers of cytosolic tangles [87,88]. Nevertheless, their nuclear localization and the conformation in paper clip originated by the phosphorylation of both epitopes [89] indicate a nuclear function in aged neurons probably related to modifications of chromatin structure [90].

Elevated content of nuclear AT100 stabilizing chromatin, especially the blocks of constitutive heterochromatin, is characteristic of aging neurons [32]. Some of these neurons abandon their quiescent state to start the cell cycle [68]. The aberrant cell-cycle reentry of these postmitotic senile neurons takes place in parallel with the presence of Lamin B2 in the nucleoplasm activating the replication sites [70]. The remaining senile neurons show a reinforced perinuclear Lamin B2 without nucleoplasmic expansions, and the presence of nuclear Tau phosphorylated at sites recognized by AT8 and AT100, which indicates the lack of an entry on S phase [91]. The phosphorylation of AT8 site induces a conformational change of Tau marked at AT100 which has been identified with inhibition of replication [92], rDNA transcriptional inactivation [93] and the preservation of this population of vulnerable AD neurons in G0 [89]. In this context, abnormal Lamin A expression establishes the border between senile cycling neurons and cycling AD neurons. The relevance of this critical step explains why Tau is considered a causal AD event [33]. The progressive decrease of nuclear Tau along I-IV AD stages strongly affects LAD and NAD stability, as the blocks of pericentromeric heterochromatin are stabilized by nuclear Tau [25]. Only the increased expression of the repressive histone mark H4K20me3 in LADs from I to IV AD stages allows preventing the complete destabilization of the heterochromatin structure. Similarly, in Tau-deprived NADs [24], H4K20me3 silences rDNA genes and stabilizes its amplification and that from nucleolar satellite DNAs [35,94]. Although this epigenetic silencing provides a survival alternative for hippocampal neurons through I-IV AD stages, the absence of H4K20me3 at AD V-VI stages indicates a total destabilization of heterochromatin blocks and global chromatin in neurons harboring NFTs [19]. Concerning B23, the displacement of this chaperone from the nucleolus to the cytoplasm, as observed in this study, has also been related to cell cycle progression [95] and in cellular stress conditions [96,97]. Further studies would help to explain the role of B23 in the cytoplasm of AD neurons.

### 3.5. Lamin A and Tau Oligomers in Neurons with NFTs

**Figure 8:**
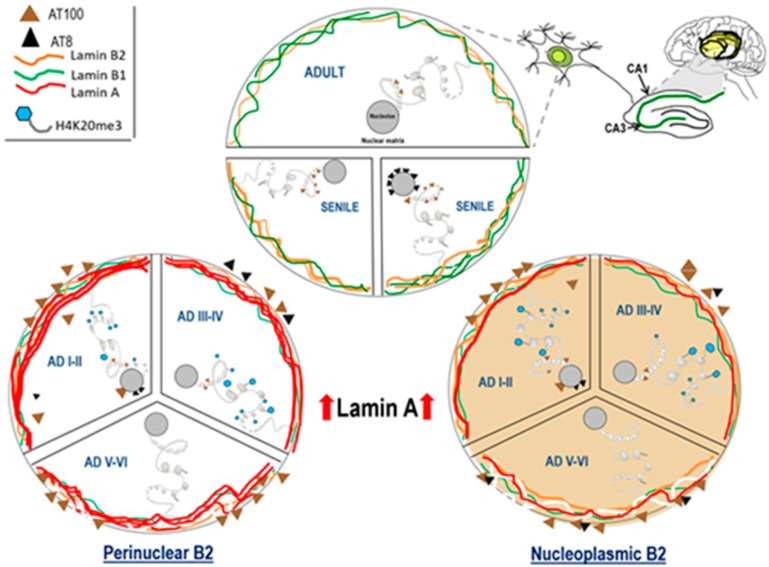
Nuclear dynamics of Tau, nuclear lamins, and H4K20me3 in the hippocampus of adult, senile AD I-II, AD III-IV, and AD V-VI Braak stages. Adult neurons have low phosphorylated Tau-AT100 expression and Lamin B1 and Lamin B2 are the components of NL. Senile neuron increment their content of phosphorylated Tau-AT100 and AT8. Changes in Lamin B2 determine the two types of neurons, those with reinforced perinuclear Lamin B2 (left) and those with nucleoplasmic Lamin B2 (right). Lamin A (left) expression is associated with H4K20me3 repressive marks in LADs and NADs. The increased presence of cytoplasmic Tau leads to hyperphosphorylation, oligomer and NFT formation.

AD start is characterized by abnormal Lamin A expression in up to 50% of hippocampal neurons, which halts them in the G1 phase preventing its further progression across the cell-cycle [98]. The addition of Lamin A to Lamins B1 and B2 gives rise to a reinforced NL that reorganizes the genome and leads to a decisive modification of the nucleus-cytoskeleton dynamics. Lamin A regulates the expression of cytoplasmic stress fibers, nuclear actin, and nuclear myosin I [99], and perhaps also Tau expression as all of them are components of the nucleo-cytoskeleton [51] and can move across the nuclear pore complex [19,33]. As a consequence of the aborted cell cycle, an excess of ectopically induced extranuclear kinases [36,64,65], hyperphosphorylate cytoplasmic Tau. In these pathological conditions, hyperphosphorylated Tau is no longer able to bind microtubules and self-associates to form prefibrillar oligomers and NFTs (Figure 2A–H). A significant fraction of hippocampal neurons that redistributed Lamin B2 to the nucleoplasm but lack Lamin A expression progress to the S phase of the cell cycle and become tetraploids [36,67]. Those neurons will progressively die during early (I-II) and intermediate (III-IV) AD stages until the late (V-VI) stages, where the number of tetraploid neurons significantly decreases [68,100]. The final balance of this process is that AD brain loss is more than 50% of their neurons, while the remaining survive thanks to Lamin A and Tau protein (Figure 8) in the form of cytoplasmic NFTs [62].

### 3.6. Methodology

The protocol was reviewed and approved by the Clinical Research Ethics Committee of the Biobanco Hospital Universitario Fundación Alcorcón (47/2018, 1 October 2018), where the neuropathological diagnosis of AD was performed. Informed consent was waived by this committee because no intervention was involved and no patient identifying information was included. Paraffin sections obtained from control and AD brains at all Braak and Braak stages, at the level of regions CA1 and CA3 of the hippocampus, were processed for immunohistochemistry and immunofluorescence. Twelve blocks from autopsied brain tissue of AD patients were divided into 3 groups of 4 cases each, ADI-II, AD III-IV, and AD V-VI. Four control cases were senile subjects in ages ranging between 65 and 68. These senile cases were compared with the 3 AD groups for quantification purposes. In addition, 4 cases of healthy adults with ages ranging between 23 and 52 years old were analyzed for some markers and also included in Lamin A quantification. The following nuclear and heterochromatin antibodies were analyzed: B23 (nucleophosmin), H4K20me3, lamins A, B1, B2, C, and phosphorylated tau (Table 1).

The immunohistochemical assessment was performed on 4 µm thick dewaxed paraffin sections. After boiling the sections in a pressure cooker with DIVA decloaking solution (Biocare Medical, LLC, Concord, CA, USA) for epitope recovery, endogenous peroxidases were blocked with Dako Peroxidase Blocking Reagent (DAKO, Glostrup, Denmark). Next, diluted primary antibodies were incubated overnight at 4 °C. After incubation with the primary antibody and PBS rinses, sections were exposed to the streptavidin-biotin marked secondary antibody. The peroxidase reaction was visualized with either 3′3-diaminobenzidine or with 3-amino-ethyl-carbazole. The sections were finally counterstained with hematoxylin (HE), dehydrated and cover-slipped for microscopic observation. The sections were observed on an Olympus microscope equipped with a digital camera (Amscope, Irvine, CA, USA). The streptavidin-biotin marked fluorescence-coupled secondary antibodies replaced secondary antibody for confocal microscopy. Nuclear acids were stained with SYTOX (Table 1).

Cell counting was performed on immunostained sections corresponding to the hippocampal regions CA1 and CA3, by a coauthor that was blind to the analyzed condition. The total number of neurons inspected (ranging from 50–60 per subject) was used to calculate the percentage of neurons exhibiting immunostaining. The percentages of neurons were obtained manually using the cell counter plugin. The contrast was enhanced, sharpness increased, and minor imperfections removed. They were then processed for quantification utilizing Fiji version 1.47t (ImageJ; Wayne Rasband, Bethesda, MD, USA). A specific built-in algorithm called color deconvolution plug-in [101] was used to separate the staining of HE and DAB into three different panels, namely, HE (panel 1), DAB only image (panel 2), and background (panel 3). Panel 2 was converted to grayscale for threshold selection. The area was selected (region of interest, ROI) by adjusting the size of the brush according to the neuron soma. From ROI, the software calculated the mean intensity of DAB and the sum of the pixel values in the ROI. All data were analyzed for normality and variance homogeneity, and several tests were used for statistical comparison. Control and AD groups were compared using the Student t-test or Mann−Whitney U test for parametric and nonparametric data, respectively. The comparison of data from multiple AD stages was performed either with one-way ANOVA followed by the Dunnett test or with Kruskal–Wallis followed by the Mann−Whitney U test to identify significant differences (* *p* < 0.05) versus the control group.

## 4. Conclusions

Cellular aging bears a close relationship with DNA damage and implies dramatic transformations of function and structure of chromatin [102]. Old postmitotic neurons try to repair their DNA by entering the cell cycle as replicative cells do. Their vast nucleo-cytoskeleton complexity, however, does not allow them to immortalize like cancer cells, and they die in the attempt [54,54,63,103]. The biological rescue mechanism of neuronal suicide is the reorganization of the nucleo-cytoskeleton through the massive expression of the highly regulated Lamin A [44] and the building of toxic NFTs (Figure 7). These dramatic changes may contribute to shut down neuronal plasticity, and trigger synaptic dysfunction, as it has been recently demonstrated in vitro [74]. Oblivion quid pro quo death.

## Figures and Tables

**Table 1:** Details of the employed antibodies.

Antibody/Supplier/Catalog Number/Manufacture	Species	Dilution
AT100 (antiphospho Tau S212-T214)/abcam/Thermo Fisher/MN1060/Waltham, MA, USA	mouse monoclonal	1:100
AT8 (antiphospho Tau S205-T205)/Thermo Fisher/MN1020/Waltham, MA, USA	mouse monoclonal	1:20
AH36 (antiphospho Tau S205-T205) Stress Marq/SMC-601/Victoria, B.C., Canada	rabbit monoclonal	1:100
Anti-Lamin A/abcam/ab26300/Cambridge, UK	rabbit polyclonal	1:500
Anti-Lamin B1/abcam/ab133741/Cambridge, UK	rabbit monoclonal	1:200
Anti-Lamin B2/abcam/ab151735/Cambridge, UK	rabbit monoclonal	1:200
Anti-Lamin C/abcam/ab125679/Cambridge, UK	rabbit polyclonal	1:20
B23 (anti-nucleophosmin)/abcam/ab10530/Cambridge, UK	mouse monoclonal	1:50
Anti- H4K20me3/abcam/ab9053/Cambridge, UK	rabbit monoclonal	1:100
Alexa –Fluor 488 secondary antibody/Life Technologies/CA, USA	goat anti-rabbit	1:300
Alexa –Fluor 633 secondary antibody/Life Technologies/Carlsbad, CA, USA	goat anti-mouse	1:200
Sytox Orange nucleic acid stain/Molecular Probes/Eugene, OR, USA		1:1000

## Supplementary Materials

The following are available online at https://www.mdpi.com/1422-0067/21/5/1841/s1.
