# Supplementary material for: Perinuclear Lamin A and Nucleoplasmic Lamin B2 Characterize Two Types of Hippocampal Neurons through Alzheimer’s Disease Progression
Source: Int J Mol Sci. 2020 Mar 7;21(5):1841. doi: 10.3390/ijms21051841 (PMC7084765; doi:10.3390/ijms21051841)
Supplement: Supplementary file 1 [file ijms-21-01841-s001.pdf]

International Journal of
Molecular Sciences

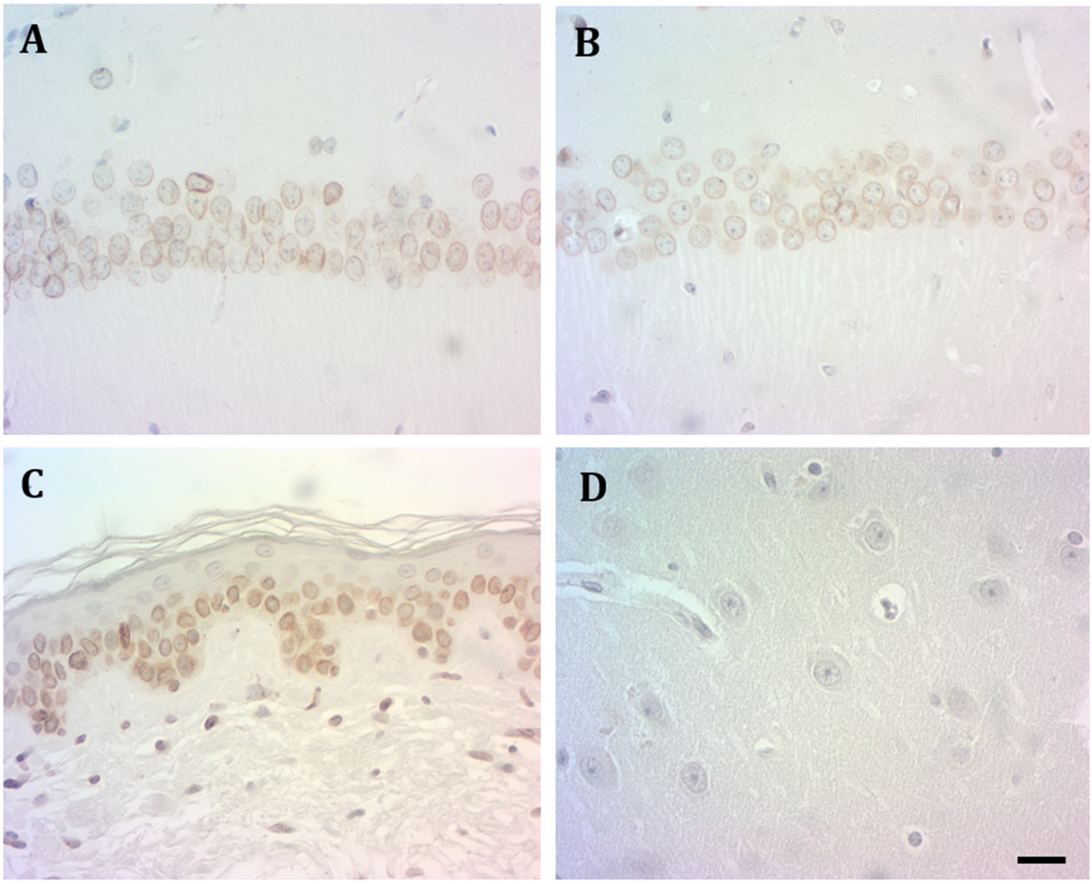

Supp. Figure S1 Lamin C. Immunopositivity to Lamin C antibody was clearly observed in the hippocampus of *wild type* mouse (A), triple transgenic AD mouse model (B) and human epidermis (C), but it was completely absent from human hippocampus (D), scale bar10 μm

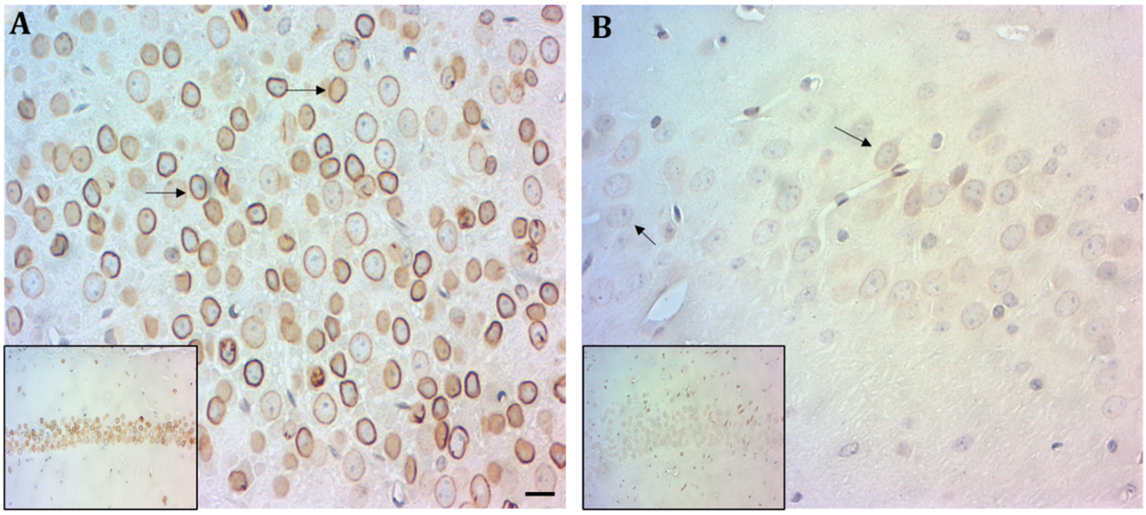

Supp. Figure S2. Test of Lamin B2 and Lamin A in the triple transgenic AD mouse model. Populations of nucleoplasmic and perinuclear Lamin B2 (A,arrows) in the CA1 region (insert). Slight perinuclear Lamin A immunopositivity (B, arrows) in the CA1 region (insert). Scale bar 10 μm
